# Quality Assessment of Physician Discharge Summaries: Instrument Development and Reliability Study

**DOI:** 10.2196/105705

**Published:** 2026-10-13

**Authors:** Leopold Bentheim, Leonard Fehring, Hendrik Friederichs, Samer Zawy Alsofy, Markus Unnewehr

**Affiliations:** 1 Department of Health Care Informatics, School of Medicine, Faculty of Health, Witten/Herdecke University, Witten, North Rhine-Westphalia, Germany; 2 Department of Medicine, Goethe University Frankfurt, Frankfurt am Main, Hessen, Germany; 3 Medical Education Research Group, Medical School OWL, Bielefeld University, Bielefeld, North Rhine-Westphalia, Germany; 4 Department of Medicine, School of Medicine, Faculty of Health, Witten/Herdecke University, Alfred-Herrhausen-Straße 50, Witten, North Rhine-Westphalia, 58455, Germany; 5 Department of Neurosurgery, St. Barbara-Klinik Hamm-Heessen, Hamm, North Rhine-Westphalia, Germany; 6 Department of Medicine, School of Medicine, Faculty of Health, Witten/Herdecke University, Alfred-Herrhausen-Straße 50, Witten, North Rhine-Westphalia, 58455, Germany, 49 2381 681 1817; 7 Department of Respiratory Medicine, Infectious Diseases, Sleep Medicine, Allergology, St. Barbara-Klinik Hamm-Heessen, Hamm, North Rhine-Westphalia, Germany

**Keywords:** discharge summaries, clinical documentation, quality assessment, instrument development, interrater reliability, care coordination, digitally supported documentation

## Abstract

**Background:**

Discharge summaries (DSs) are key clinical documents at the interface between inpatient and outpatient care. Their quality affects continuity of care, coordination, and patient safety, yet information transfer at discharge is often incomplete, delayed, or unclear. Standardized instruments for assessing DS quality remain scarce.

**Objective:**

This study aimed to develop a structured, recipient-oriented quality assessment instrument (QAI) for physician DS as a pragmatic measure of discharge communication quality and to conduct an initial evaluation of its applicability and interrater reliability.

**Methods:**

We conducted a formative instrument development study using a structured, iterative author panel consensus process informed by Delphi principles, condensing 43 recipient-oriented DS criteria into 12 items across 3 domains: medical content, structure and organization, and language and clarity. It was applied in a pragmatic pilot reliability evaluation to 20 anonymized internal medicine DSs, independently assessed by 5 physician raters with heterogeneous clinical backgrounds who were not involved in QAI development. Interrater reliability was evaluated using Krippendorff alpha and quadratically weighted Gwet agreement coefficient 2 (AC2). Reporting followed the Guidelines for Reporting Reliability and Agreement Studies.

**Results:**

The median overall quality score was 78.5% (IQR 75.3%-82.5%), and the mean was 79.1% (SD 5.0%). Scores were highest for language and clarity and lowest for medical content. Overall interrater reliability differed by coefficient: Krippendorff α was 0.45 (95% CI 0.34-0.55) whereas Gwet AC2 was 0.76 (95% CI 0.70-0.80). The largest discrepancy was observed for language and clarity, with an α of 0.02 (95% CI −0.06 to 0.10) and an AC2 of 0.88 (95% CI 0.84-0.91).

**Conclusions:**

The QAI provides preliminary evidence for a clinically applicable approach to assessing key quality dimensions of physician DS. Interrater reliability varied across domains and reliability coefficients, with a marked discrepancy between AC2 and Krippendorff alpha for language and clarity. These findings support its use for exploratory quality improvement and future validation.

## Introduction

Discharge summaries (DSs) are central physician-to-physician documents in transitional care, communicating diagnoses, key findings, therapeutic interventions, medication changes, and recommendations for ongoing care [1]. However, information transferred at discharge is often incomplete, delayed, or unclear [1,2]. Delayed dissemination, missing information on diagnoses, medication, pending results, or follow-up recommendations, and inconsistent presentation may impair continuity of care and patient safety [3-7].

The quality of DS is influenced not only by individual writing practices but also by system-level factors, including workload and time pressure, electronic documentation systems and templates, institutional workflows, and the availability of clinical information at the time of discharge [8].

From a formative health services research perspective, DS quality represents a measurable component of care coordination at the hospital-outpatient interface and may serve as a target for iterative quality improvement, audit and feedback, and digitally supported documentation processes.

Recipient-oriented studies indicate that primary care physicians value clear structure, complete clinical information, concise synthesis of the hospital course, and specific recommendations for further management [9-12]. Accordingly, DS quality should be assessed not only by formal completeness but also by usefulness for the recipient.

Despite increasing emphasis on standardized discharge documentation, concise and reproducible instruments for structured quality assessment remain limited. Several instruments have been developed to assess the quality of DS or physician documentation. These include point-based DS scoring systems, tools focusing on specific groups such as residents, assessment approaches oriented toward general practitioners, and more general documentation quality instruments. However, existing approaches differ substantially in their intended use, item selection, weighting, and validation strategy. To our knowledge, no previous DS assessment instrument has combined recipient-derived quality criteria with explicit weighting based on empirically measured recipient priorities across the domains of medical content, structure and organization, and language and clarity [13-17].

Building on the 43 recipient-oriented criteria identified by Unnewehr et al [10], this study aimed to develop a concise and clinically usable quality assessment instrument (QAI) for physician DS and to conduct an initial evaluation of its applicability and interrater reliability in internal medicine DS as an exploratory quality-of-care measurement approach.

## Methods

This methodological study comprised 2 parts: development of a structured QAI for physician DS and an assessment of its applicability and interrater reliability in anonymized internal medicine DS. Reporting was informed by the Guidelines for Reporting Reliability and Agreement Studies [18].

### Conceptual Considerations

The QAI was designed to evaluate clinically relevant dimensions of DS quality, including medical content, structure and organization, and language and clarity. These domains should be interpreted as complementary components of document quality rather than as indicators of a single latent construct. Accordingly, the total score is a pragmatic summary indicator that supports structured comparison and feedback but does not by itself establish psychometric validity.

The instrument was therefore conceived as a pragmatic quality assessment tool for health services research and local quality improvement, not as a diagnostic or patient-level outcome measure.

### Development of the QAI

#### Overview

The QAI was conceptually based on Unnewehr et al [10], who identified 43 recipient-oriented quality criteria for DS. Development followed a structured, iterative consensus process within the author panel, informed by Delphi principles of item generation, item reduction, and consensus building [18-21]. The process was not a formal Delphi study, as it did not involve an independent and anonymous expert panel. The aim was to translate the 43 criteria into a shorter instrument for pragmatic use by physicians. Consensus thresholds were used as pragmatic guidance rather than as formal validation criteria.

#### Step 1: Assessment of Relevance and Practical Assessability

Each of the 43 criteria was reviewed for clinical relevance, clarity, and feasibility of assessment based on the DS alone. Criteria considered to be of limited direct relevance for clinical decision-making, predominantly administrative, or difficult to assess reliably from the document itself were marked for exclusion or merging. This feasibility-oriented review was conducted by the author panel as part of the structured, iterative consensus process. The subsequent raters were not involved in instrument development.

#### Step 2: Identification of Overlap and Item Reduction

The remaining criteria were examined for semantic similarity and conceptual overlap. Related criteria were merged into broader items where appropriate, whereas clearly defined, clinically relevant, and distinct criteria were retained as stand-alone items.

#### Step 3: Consensus Refinement, Pilot Testing, and Final Adjustment

The preliminary item set was iteratively refined within the author panel with regard to wording, structure, scoring logic, and practical applicability, and was pilot-tested on 6 DSs. These pilot documents constituted a separate pilot sample and were not part of the sampling frame used for the subsequent reliability analysis.

This process resulted in the exclusion of 12 original criteria and the condensation of the remaining 31 criteria into 12 items assigned to 3 domains: medical content, structure and organization, and language and clarity (Figure 1).

**Figure 1.**
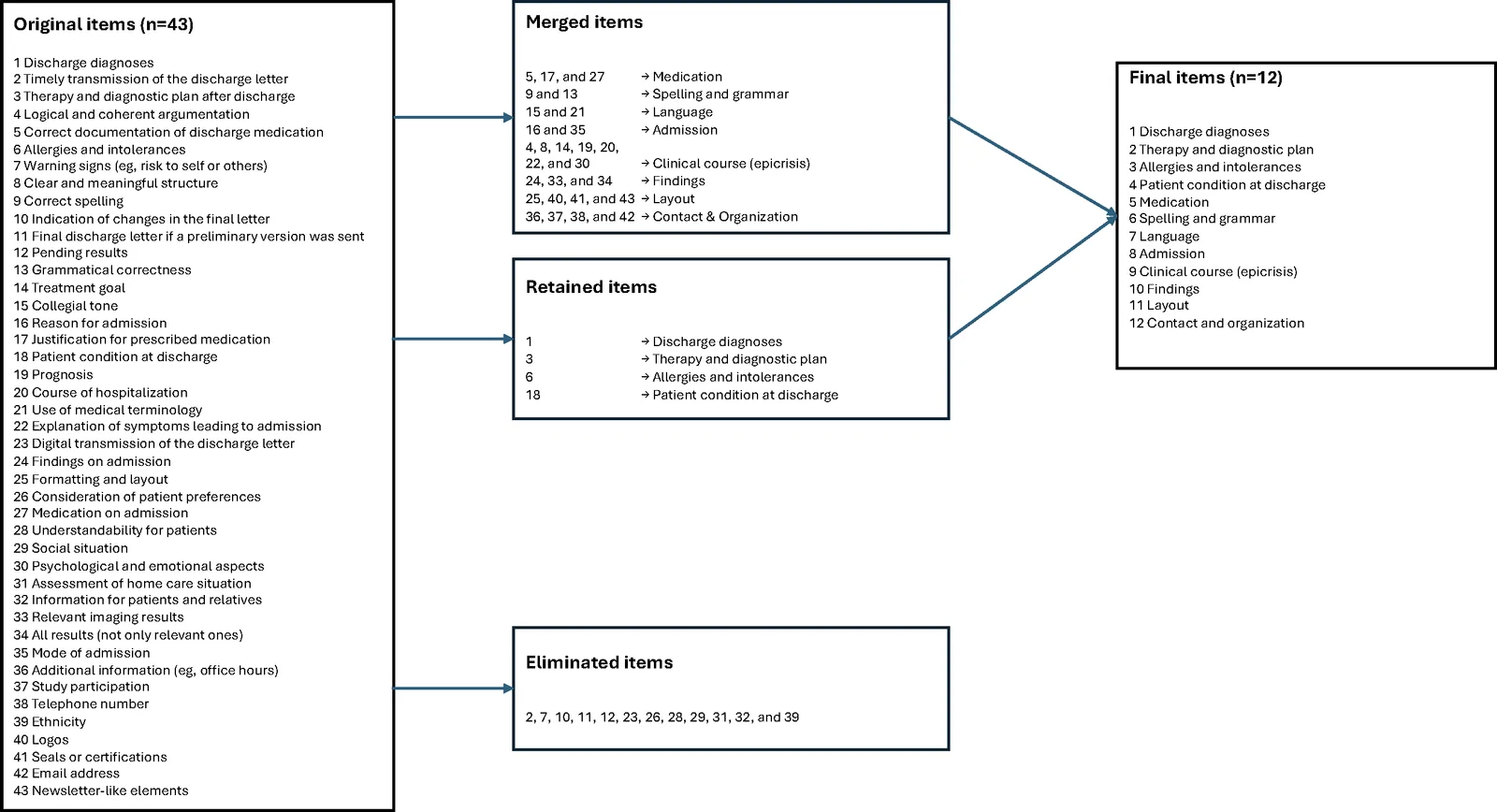
Structured, iterative author panel consensus process.

A complete crosswalk documenting which of the original 43 criteria were retained, merged, or excluded is provided in Multimedia Appendix 1.

Detailed item definitions and scoring instructions for the final QAI items are provided in Multimedia Appendix 2.

### Weighting and Score Calculation

Each item was rated on a 5-point Likert scale ranging from 0 to 4 [22].

Items were not equally weighted. Weighting was based on Unnewehr et al [10], specifically the mean, representing perceived relevance, and the SD, reflecting agreement among experts.

For items created by merging multiple original criteria, the mean was calculated as the arithmetic mean of the corresponding criterion-specific means, while the SD was aggregated as the root mean square of the corresponding criterion-specific SDs. This pragmatic aggregation preserved the contribution of the original criteria when respondent-level covariance data were not available. The resulting item weight was calculated as mean×(1/SD), and the weighted item score as rating×mean×(1/SD).

### Handling of Nonapplicable Items

If an item could not be assessed, it was marked as nonapplicable (NA) items, excluded from score calculation, and the maximum achievable score was recalculated based only on rated items. The total score for each DS was calculated as the sum of all weighted item scores and expressed as a percentage of the maximum achievable score. Domain-specific scores were calculated accordingly.

The frequency of NA ratings was additionally summarized by item and rater and is reported in Multimedia Appendix 3.

### Sample of DSs

A total of 20 DSs were selected using a predefined stratified random sampling procedure. The sampling frame consisted of all physician DSs received by the 3 participating general practices between January 1, 2025, and December 31, 2025, that fulfilled predefined eligibility criteria: discharge after an inpatient stay of at least 1 night, internal medicine as the primary specialty, availability of the complete physician DS, and suitability for full anonymization. Duplicate documents, preliminary discharge notes, outpatient reports, emergency department notes without inpatient admission, and documents from noninternal medicine specialties were excluded.

Within each practice, eligible DSs were identified by the local practice team and assigned consecutive local identification numbers. The research team had no access to patient-identifying information. A stratified random sample was drawn separately within each practice to ensure representation from rural, small town, and urban settings. Selection was performed without replacement using a computer-generated random sequence, with the sample distributed approximately equally across the 3 practices.

Across the 3 practices, 47 eligible DSs were identified, of which 20 (42.6%) were selected: 7 of 12 (58.3%) in the rural practice, 6 of 20 (30%) in the small town practice, and 7 of 15 (46.7%) in the urban practice. The 20 selected DSs originated from 12 different hospitals. Six hospitals contributed 1 DS each, 4 hospitals contributed 2 DSs each, and 2 hospitals contributed 3 DSs each.

After selection, the DSs were anonymized before transfer. If a selected document was incomplete, duplicated, or not fully anonymizable, it was replaced by the next document on the pregenerated random list. No documents were excluded on the basis of perceived quality, length, hospital of origin, diagnoses, or expected rating difficulty.

This practice-based sampling approach was intended to reflect DSs as they are received and used in routine outpatient care, rather than as documents selected from a single hospital information system.

### Raters

All 20 DSs were assessed by 5 physicians with routine clinical experience in writing, receiving, or interpreting DSs. The raters represented both inpatient and outpatient perspectives and had heterogeneous clinical backgrounds, including 1 resident physician, 2 general practitioners, 1 chief physician, and 1 professor of medicine. This heterogeneity was intentional, as the QAI was designed to assess physician-to-physician discharge communication across the inpatient-outpatient interface rather than specialty-specific aspects of internal medicine.

The raters were recruited from the professional networks of the authors based on their clinical experience with DSs and their interest in discharge communication and documentation quality. None of the raters had an existing professional working relationship with members of the author group, and none had been involved in the item development process, including the selection, reduction, or consolidation of the 43 source criteria into the final 12 QAI items.

Before assessment, all raters received the final instrument, including item definitions and scoring instructions. No formal calibration session or consensus training was conducted. The raters received only anonymized DSs labeled with study identification numbers, were blinded to patient identity, practice and hospital of origin, and institutional identifiers, and rated independently without discussion or case-specific guidance.

### Statistical Analysis

Interrater reliability was assessed using Krippendorff alpha and Gwet agreement coefficient 2 (AC2) with quadratic weighting. The reliability analysis was conducted at the level of individual case item units, defined as the combination of a DS and a specific item.

Krippendorff alpha was calculated using an ordinal metric with quadratic distance weighting. Gwet AC2 was included as a complementary measure because it is considered more robust in the presence of skewed category distributions and high agreement [23,24]. Reliability was also assessed for the predefined domains.

Missing ratings due to NA classifications were treated as missing values and did not constitute a separate category. Case item units were retained as long as at least 2 valid ratings were available.

CIs (95%) were estimated using a bootstrap approach at the case level.

Descriptive analyses reported the median and IQR as primary measures, with the mean and SD as secondary measures. Statistics were based on unweighted averages of available ratings per case item unit, transformed to percentages and aggregated at the DS level.

To assess the influence of the weighting procedure on the resulting QAI scores, a sensitivity analysis compared weighted and unweighted scores at the DS level. For the unweighted analysis, all QAI items contributed equally to the overall and domain-specific scores. Weighted and unweighted overall scores were compared descriptively, including their mean difference and Pearson correlation, and changes in domain-specific scores and domain ranking were examined.

### Methodological Considerations on Validity

As this study represents an exploratory methodological evaluation, the primary focus was on feasibility, applicability, and interrater reliability rather than on comprehensive validity evidence. Interrater reliability was prioritized because reproducible application across independent assessors was considered a prerequisite for subsequent evaluation of further measurement properties and broader practical use.

Content validity was supported indirectly by previously published recipient-oriented quality criteria and the structured, iterative clinical consensus within the author group but was not further assessed using quantitative content validity indices or cognitive interviews. COSMIN (Consensus-Based Standards for the Selection of Health Measurement Instruments)–related methodological work emphasizes content validity and rigorous assessment of measurement properties, although COSMIN was originally developed mainly for health status and patient-reported outcome instruments [25-27].

### Ethical Considerations

This study was conducted as a quality improvement study based on anonymized hospital DSs generated during routine clinical care. No patients were contacted, no intervention was performed, and no directly identifiable patient data were analyzed or reported.

The study was reviewed by the ethics committee of the *Universität Witten/Herdecke*, Germany, which confirmed that formal ethics approval and individual informed consent were not required for this analysis of anonymized documents (S-193/2025).

All methods were carried out in accordance with the Declaration of Helsinki and applicable data protection regulations.

## Results

The final set of items after the structured consensus process, including weighting parameters, is shown in Table 1.

**Table 1.** Final set of items after the structured consensus process.

Item number	Items	Rating, mean (SD)	Weight (mean value/SD)	Maximum score contribution (%)
1	Discharge diagnoses	4.91 (0.31)	7.80	15.95
2	Therapy and diagnostic plan after discharge	4.79 (0.42)	6.85	14
3	Allergies and intolerances	4.71 (0.49)	4.84	9.89
4	Patient condition at discharge	4.50 (0.64)	4.08	8.33
5	Medication	4.45 (0.63)	4.02	8.21
6	Spelling and grammar	4.49 (0.60)	3.68	7.52
7	Language	4.43 (0.65)	3.56	7.27
8	Admission	4.28 (0.74)	3.32	6.78
9	Clinical course (epicrisis)	4.47 (0.61)	4.12	8.43
10	Findings	4.14 (0.76)	2.66	5.44
11	Layout	3.41 (0.94)	2.14	4.37
12	Contact and organization	3.67 (0.91)	1.87	3.82

A total of 20 DSs were evaluated by 5 independent raters using the QAI. The characteristics of the raters and their mean overall QAI scores are presented in Table 2. Overall, the analysis showed favorable quality ratings across the assessed documents. The median overall score was 78.5% (IQR 75.3%-82.5%), and the mean overall score was 79.1% (SD 5.0%).

**Table 2.** Characteristics of the raters and their mean overall quality assessment instrument (QAI) scores.

Raters	Professional role	Clinical setting	Experience with discharge summaries	Medical specialty	Overall QAI score (%), mean (SD)
Rater 1	Resident physician	Inpatient	Routine use (>6 years)	Neurosurgery	74.83 (10.12)
Rater 2	General practitioner	Outpatient	Extensive experience (>15 years)	General practice	76.85 (7.31)
Rater 3	General practitioner	Outpatient	Extensive experience (>25 years)	General practice	87.30 (7.94)
Rater 4	Chief physician	Inpatient	Extensive experience (>20 years)	Pulmonology	82.97 (8.75)
Rater 5	Professor of medicine	Inpatient	Extensive experience (>20 years)	General medicine	74.79 (9.44)

Differences were observed across domains. The highest ratings were found for language and clarity, with a median of 87.5% (IQR 85.0%-90.0%) and a mean of 86.8% (SD 4.6%). Structure and organization showed intermediate values, with a median of 80.0% (IQR 74.4%-85.0%) and a mean of 80.0% (SD 9.6%). The lowest ratings were observed for medical content, with a median of 75.6% (IQR 73.3%-81.7%) and a mean of 77.0% (SD 7.2%; Table 3).

**Table 3.** Descriptive results of the quality assessment instrument.

Domains	Rating (%), median (IQR)	Rating (%), mean (SD)
Medical content	75.6 (73.3-81.7)	77.0 (7.2)
Structure and organization	80.0 (74.4-85.0)	80.0 (9.6)
Language and clarity	87.5 (85.0-90.0)	86.8 (4.6)
Overall score	78.5 (75.3-82.5*)*	79.1 (5.0*)*

Mean overall scores differed between individual raters, ranging from 74.79% to 87.30% (rater 1: 74.83%; rater 2: 76.85%; rater 3: 87.30%; rater 4: 82.97%; and rater 5: 74.79%; Table 2). The difference between the lowest and highest mean rater score was 12.51 percentage points.

Thus, the instrument distinguished between formal linguistic quality and clinically actionable content, a distinction that is relevant for quality improvement in discharge communication.

In the sensitivity analysis, the mean overall score was 77.3% using the weighted scoring procedure compared with 79.1% using equal item weighting, corresponding to a mean difference of −1.8 percentage points. Weighted and unweighted overall scores were strongly correlated (Pearson *r*=0.975). The maximum absolute difference at the individual DS level was 4.7 percentage points. Mean domain scores were 75.2% vs 77.0% for medical content, 79.5% vs 80.0% for structure and organization, and 86.8% vs 86.8% for language and clarity for weighted and unweighted scoring, respectively. The ranking of the 3 domains was unchanged.

Across the 1200 possible item ratings, 15 (1.25%) were classified as NA. Item- and rater-specific NA frequencies are provided in Multimedia Appendix 3.

Interrater reliability estimates varied depending on the statistical coefficient and domain assessed. For the overall rating, Gwet AC2 showed good agreement (0.76, 95% CI 0.70-0.80) and Krippendorff α showed moderate agreement (0.45, 95% CI 0.34-0.55; Table 4).

**Table 4.** Interrater reliability.

Domains	Krippendorff α (95% CI)	Gwet AC2 (95% CI)
Overall score	0.45 (0.34 to 0.55)	0.76 (0.70 to 0.80)
Medical content	0.49 (0.36 to 0.60)	0.72 (0.64 to 0.78)
Structure and organization	0.28 (0.18 to 0.36)	0.78 (0.70 to 0.84)
Language and clarity	0.02 (−0.06 to 0.10)	0.88 (0.84 to 0.91)

Domain-specific analyses showed varying agreement. For medical content, Gwet AC2 was 0.72 (95% CI 0.64-0.78) and Krippendorff α was 0.49 (95% CI 0.36-0.60). For structure and organization, AC2 was 0.78 (95% CI 0.70-0.84), and α was 0.28 (95% CI 0.18-0.36). The highest agreement was observed for language and clarity, with an AC2 of 0.88 (95% CI 0.84-0.91), whereas Krippendorff α was 0.02 (95% CI −0.06 to 0.10).

These findings indicate that reliability was not uniform across domains. In particular, the low alpha for language and clarity suggests that high observed agreement and high AC2 should be interpreted cautiously, possibly in the context of restricted variance, ceiling effects, and limited discriminative capacity.

## Discussion

### Principal Findings

This study describes the development and methodological evaluation of a structured instrument for assessing physician DSs as a quality dimension of transitional care in internal medicine.

The findings suggest that the QAI is feasible for pragmatic clinical use and yields acceptable agreement, although reliability varied across domains.

To account for expert consensus, a weighting factor based on the inverse SD (1/SD) was applied, giving greater weight to items with higher agreement. This approach allows both perceived relevance and expert agreement to be incorporated into the scoring system and provides an exploratory, more differentiated representation of DS quality [18,19]. In the sensitivity analysis, weighted and unweighted overall scores were strongly correlated, with a mean difference of −1.8 percentage points, and the ranking of the 3 domains remained unchanged. This suggests that the observed domain pattern was not primarily driven by the weighting procedure.

### Interpretation in Relation to Previous Research

The conceptual basis of the instrument is closely aligned with Unnewehr et al [10], who emphasized recipient-oriented quality criteria such as completeness, clarity, and relevance for further care. Previous approaches to assessing DS quality differ substantially in their scope, conceptual basis, and methodological design. Rao et al [13] developed a point-based scale covering 4 quality domains, whereas Hommos et al [15] proposed an instrument specifically for evaluating residents’ DSs and examined its reliability using generalizability analysis. Savvopoulos et al [17] developed a weighted DS quality scoring tool based on a literature review, stakeholder consensus, and interrater testing. The Physician Documentation Quality Instrument–9 (PDQI-9) provides a measure of general physician documentation quality and has also been applied to DSs but was not designed specifically for the hospital-to-outpatient transition [14]. Mahfouz et al [16] developed a DS assessment tool based on components considered important by general practitioners, although this primarily measures recipients’ satisfaction with discharge information rather than the quality of individual documents. Recent work has also proposed standardized approaches for specific DS components, particularly the diagnosis section, based on physician preferences and consensus-derived content elements [7].

Compared with these approaches, the present study extends this line of work by introducing a concise, multidimensional assessment framework across several domains of discharge communication. A distinctive feature of the QAI is that both its item selection and weighting are directly linked to empirically measured priorities of physicians receiving DSs. It thereby integrates recipient-derived priorities across medical content, structure and organization, and language and clarity within a single DS-specific assessment framework. The QAI should therefore be regarded as a complementary approach requiring further validation rather than as a replacement for existing instruments.

The descriptive results showed generally favorable ratings, with the highest scores for language and clarity and the lowest scores for medical content. This pattern may indicate that formal and linguistic aspects of DSs are more consistently achieved than clinically detailed and actionable content. A DS may be well written and clearly structured while still lacking information relevant for safe follow-up care.

Importantly, variation in QAI scores should not be attributed solely to the documentation skills of individual physicians. As DS quality emerges within a broader organizational and technical context, the QAI primarily assesses the quality of the resulting document rather than individual physician performance. This distinction is particularly relevant when using the instrument for quality improvement or comparisons between settings.

Because the sample was small and drawn from only 3 practices, these findings should be interpreted as an initial application in a pragmatic practice-based sample rather than as findings that can be generalized to DSs more broadly. The 20 selected DSs originated from 12 different hospitals, limiting the influence of documentation practices from any single institution and increasing the heterogeneity of the pragmatic sample.

### Interpretation of Reliability Findings

The analysis used 2 complementary reliability coefficients, which allowed a more nuanced interpretation of agreement patterns than reliance on a single statistic. Krippendorff alpha yielded lower reliability estimates than Gwet AC2 overall and across domains. Such discrepancies may occur in the presence of skewed category distributions, high agreement, or restricted variance, conditions under which Gwet AC2 is considered more robust [23,24,28].

The “high agreement but low kappa” paradox describes a situation in which high observed agreement may be accompanied by low kappa values when marginal distributions are markedly imbalanced [28]. In the present study, this discrepancy was particularly evident for language and clarity, for which Gwet AC2 was 0.88, whereas Krippendorff α was 0.02. Restricted variance and ceiling effects may partly explain this marked discrepancy, but the near-zero alpha also indicates limited reliability according to this coefficient and may reflect limited discriminative capacity or insufficiently distinct rating anchors.

The individual mean overall scores also showed differences in rating levels between raters, ranging from 74.79% to 87.30%. This variation indicates that individual raters may apply the QAI criteria with different levels of stringency. Given the small number of raters and their heterogeneous clinical backgrounds, these differences cannot be attributed to specific professional characteristics. The absence of formal rater training or calibration may also have contributed to this variability.

Therefore, the present study does not establish robust reliability across all domains. Instead, it identifies areas that may require refinement, including clearer item definitions, more specific rating anchors, testing in more heterogeneous samples, and comparison of different scoring approaches.

### Rater Composition and Training

None of the 5 raters had been involved in the item development process, including the selection, reduction, or consolidation of the 43 source criteria into the final 12 QAI items. Although all assessments were performed independently without discussion or case-specific guidance, the composition of the rater group may limit the generalizability of the reliability estimates to fully external and instrument-naïve users. Future validation should therefore include a larger and fully external rater sample.

The absence of formal rater training may have contributed to variability, particularly in domains requiring more subjective judgment. Future studies should examine whether standardized rater training, calibration examples, or revised item anchors improve interrater reliability.

### Implications for Health Services Research and Quality Improvement

In general internal medicine, patients are often affected by multimorbidity, medication changes, pending diagnostics, and the need for close coordination with primary care after hospitalization. In this context, a structured instrument may support local quality assurance and targeted improvement of discharge practice, consistent with the broader importance of structured communication tools in complex patient populations [1,29,30].

For health services research, the QAI may be useful because it operationalizes discharge communication quality at the interface between hospitals and primary care. This may support comparative analyses of documentation formats, evaluation of quality improvement interventions, and the development of feedback systems within learning health care organizations.

Potential applications include local audit and feedback, educational assessment of discharge documentation, comparison of documentation formats, evaluation of quality improvement interventions, and future evaluation of digitally supported or AI-assisted discharge documentation workflows. However, the present reliability estimates are based on ratings by 5 physicians and do not establish the reliability of the QAI when used by a single assessor. Accordingly, these applications should remain exploratory and preferably involve multiple raters or standardized calibration until single-rater reliability has been specifically evaluated. Use for high-stakes benchmarking or performance assessment would be premature at this stage.

### Limitations

The QAI development process was structured and empirically informed but did not include additional formal external validation procedures such as an independent expert panel, quantitative content validity assessment, or cognitive pretesting. In addition, the initial item reduction and refinement process was conducted within the author group rather than by an independent external panel. Although the instrument was grounded in previously published recipient-derived criteria, the absence of independent panelists and anonymous consensus rounds limits the strength of the content validity evidence and should be addressed in subsequent validation studies.

Only internal medicine DSs were included. Generalizability to surgical disciplines, emergency care, specialized inpatient settings, other health care systems, and non-German care contexts remains unknown. However, the underlying problem of discharge communication across care settings is not specific to the German health care system, supporting the relevance of further testing in other contexts.

The sample was drawn from DSs available in 3 participating general practices and should be interpreted as a pragmatic practice-based sample rather than as a population-representative sample. Accordingly, the study should be interpreted as an initial methodological evaluation focused on feasibility and interrater reliability rather than as evidence of population-level DS quality.

Finally, the study did not assess construct validity, criterion validity, responsiveness, discriminative capacity, intrarater reliability, or associations with clinical outcomes, recipient satisfaction, avoidable follow-up problems, or patient safety indicators. Future studies should address these measurement properties in larger and more diverse samples.

### Conclusions

The QAI described in this study provides a structured and clinically practical approach for assessing physician DSs as a quality dimension of transitional care. In this initial evaluation, the instrument showed moderate to good interrater agreement depending on the reliability metric applied.

These findings support further validation in larger and more diverse samples, including other health care settings and a dedicated evaluation of reliability when applied by a single assessor.

The QAI should not yet be interpreted as a fully validated measurement instrument. Its potential use for exploratory quality assessment, educational feedback, and evaluation of digitally supported discharge documentation requires further validation, and use for high-stakes benchmarking is not yet supported.

## Supplementary material

10.2196/105705 Multimedia Appendix 1 Crosswalk of the 43 original recipient-oriented criteria to the final 12 quality assessment instrument (QAI) items.

10.2196/105705 Multimedia Appendix 2 Item definitions of the quality assessment instrument (QAI).

10.2196/105705 Multimedia Appendix 3 Nonapplicable (NA) items.

## Abbreviations

AC2: agreement coefficient 2
COSMIN: Consensus-Based Standards for the Selection of Health Measurement Instruments
DS: discharge summary
NA: nonapplicable
PDQI-9: Physician Documentation Quality Instrument–9
QAI: quality assessment instrument
