# Supplementary material for: Quality Assessment of Physician Discharge Summaries: Instrument Development and Reliability Study
Source: JMIR Form Res. 2026 Oct 13;10:e105705. doi: 10.2196/105705 (PMC13660460; doi:10.2196/105705)
Supplement: Supplementary file 1 [file formative-v10-e105705-s001.pdf]

Multimedia Appendix 1 Crosswalk from the 43 original recipient-oriented criteria to the final 12 QAI items

No.	Original criterion	Development decision	Final QAI item
1	Discharge diagnoses	Retained	Discharge diagnoses
2	Timely transmission of the discharge letter	Eliminated	—
3	Therapy and diagnostic plan after discharge	Retained	Therapy and diagnostic plan after discharge
4	Logical and coherent argumentation	Merged	Clinical course (epicrisis)
5	Correct documentation of discharge medication	Merged	Medication
6	Allergies and intolerances	Retained	Allergies and intolerances
7	Warning signs (e.g., risk to self or others)	Eliminated	—
8	Clear and meaningful structure	Merged	Clinical course (epicrisis)
9	Correct spelling	Merged	Spelling & grammar
10	Indication of changes in the final letter	Eliminated	—
11	Final discharge letter if a preliminary version was sent	Eliminated	—
12	Pending results	Eliminated	—
13	Grammatical correctness	Merged	Spelling & grammar
14	Treatment goal	Merged	Clinical course (epicrisis)
15	Collegial tone	Merged	Language
16	Reason for admission	Merged	Admission
17	Justification for prescribed medication	Merged	Medication
18	Patient condition at discharge	Retained	Patient condition at discharge
19	Prognosis	Merged	Clinical course (epicrisis)
20	Course of hospitalization	Merged	Clinical course (epicrisis)
21	Use of medical terminology	Merged	Language

No.	Original criterion	Development decision	Final QAI item
22	Explanation of symptoms leading to admission	Merged	Clinical course (epicrisis)
23	Digital transmission of the discharge letter	Eliminated	—
24	Findings on admission	Merged	Findings
25	Formatting and layout	Merged	Layout
26	Consideration of patient preferences	Eliminated	—
27	Medication on admission	Merged	Medication
28	Understandability for patients	Eliminated	—
29	Social situation	Eliminated	—
30	Psychological and emotional aspects	Merged	Clinical course (epicrisis)
31	Assessment of home care situation	Eliminated	—
32	Information for patients and relatives	Eliminated	—
33	Relevant imaging results	Merged	Findings
34	All results (not only relevant ones)	Merged	Findings
35	Mode of admission	Merged	Admission
36	Additional information (e.g., office hours)	Merged	Contact & organization
37	Study participation	Merged	Contact & organization
38	Telephone number	Merged	Contact & organization
39	Ethnicity	Eliminated	—
40	Logos	Merged	Layout
41	Seals or certifications	Merged	Layout
42	Email address	Merged	Contact & organization
43	Newsletter-like elements	Merged	
