# Supplementary material for: Quality Assessment of Physician Discharge Summaries: Instrument Development and Reliability Study
Source: JMIR Form Res. 2026 Oct 13;10:e105705. doi: 10.2196/105705 (PMC13660460; doi:10.2196/105705)
Supplement: Supplementary file 2 [file formative-v10-e105705-s002.pdf]

Multimedia Appendix 2. Item definitions of the QAI

Item No.	Item	Definition
1	**Discharge diagnoses**	The discharge summary provides a complete, logically structured, and medically accurate list of all relevant diagnoses at discharge. The diagnosis list contains diagnoses only or, in exceptional cases, findings with diagnostic equivalence (e.g., “dyspnea of unclear origin”), and excludes therapies, discussions, or recommendations. Main and secondary diagnoses are clearly labeled, meaningfully structured according to pathological entities and causal relationships, and, where appropriate, include staging or severity grading.
2	**Therapy and diagnostic plan after discharge**	Recommendations for further treatment and diagnostics are complete, specific, and clearly addressed to the recipient. It is clearly indicated which measures should be performed, when, and by whom. If no further plan is required, the item is rated as not applicable (N/A).
3	**Allergies and intolerances**	Relevant allergies, intolerances, or warning information are completely documented and clearly labeled to ensure patient safety. If no allergies are present, this is explicitly stated. If no information is provided, the criterion is considered not fulfilled.
4	**Patient condition at discharge**	The current clinical condition of the patient at discharge is clearly described and is consistent with the stated diagnoses, therapies, and recommendations.
5	**Medication**	Discharge medication is complete, correct, and consistently presented. Changes compared to admission are clearly indicated and justified. Consistency with diagnoses and the medication plan is ensured.
6	**Spelling & grammar**	The text is free from orthographic and grammatical errors. Language and syntax are appropriate for the medical context.

Item No.	Item	Definition
**7**	**Language**	The wording is precise, medically accurate, and appropriate for the intended recipient. The text avoids ambiguity, colloquial language, unnecessary abbreviations, and redundancy.
**8**	**Admission**	Information regarding admission (reason for admission, symptoms, prior findings, initial assessment, and mode of admission) is presented completely and in a comprehensible manner.
**9**	**Clinical course (epicrisis)**	The epicrisis provides a structured and comprehensive summary of the inpatient stay, including diagnoses, clinical course, and treatment outcomes. It addresses the initial clinical question, describes the therapeutic goals and their achievement, summarizes the medical reasoning of the case, and evaluates diagnostic findings.
**10**	**Findings**	Relevant diagnostic findings (laboratory results, imaging, functional tests) are presented completely, correctly, and in a concise manner. Normal findings are explicitly indicated as such, and irrelevant details are omitted.
**11**	**Layout**	The discharge summary has a clear, consistent, and well-structured layout. Paragraphs, headings, and formatting enhance readability and comprehensibility. The document is free of promotional or institutionally irrelevant elements and maintains a professional and objective tone.
**12**	**Contact and organization**	Organizational and contact information relevant for further care (e.g., responsible departments, contact persons) is clearly presented and facilitates continuity of care.
