# Supplementary material for: Quality Assessment of Physician Discharge Summaries: Instrument Development and Reliability Study
Source: JMIR Form Res. 2026 Oct 13;10:e105705. doi: 10.2196/105705 (PMC13660460; doi:10.2196/105705)
Supplement: Supplementary file 3 [file formative-v10-e105705-s003.pdf]

Multimedia Appendix 3. Non-applicable (N/A) items. Across 1,200 possible item ratings, 15 (1.25%) were classified as N/A.

Item	Rater 1	Rater 2	Rater 3	Rater 4	Rater 5	Total N/A
Discharge diagnoses	0	0	0	0	0	**0**
Therapy and diagnostic plan after discharge	1	1	0	0	1	**3**
Allergies and intolerances	1	0	0	3	2	**6**
Patient condition at discharge	1	1	0	0	1	**3**
Medication	1	0	0	0	0	**1**
Spelling & grammar	0	0	0	0	0	**0**
Language	0	0	0	0	0	**0**
Admission	0	0	0	0	0	**0**
Clinical course (epicrisis)	0	0	0	0	0	**0**
Findings	0	0	0	0	0	**0**
Layout	0	0	0	0	0	**0**
Contact & organization	0	0	2	1	0	**2**
**Total**	**3**	**2**	**2**	**4**	**4**	**15**
